# A Staged Approach From Venoarterial Extracorporeal Membrane Oxygenation (V-A ECMO) to ProtekDuo Circuit for Right Ventricular Support in Cardiogenic Shock: A Case Report

**DOI:** 10.7759/cureus.83908

**Published:** 2025-05-11

**Authors:** Zi Wei Liao, Jeffrey S Peterson, Kaitlin Tarr, Lovkesh Arora

**Affiliations:** 1 Anesthesia, University of Iowa Health Care, Iowa City, USA; 2 Cardiology, University of Iowa Health Care, Iowa City, USA

**Keywords:** cardiogenic shock, ecmo, mechanical circulatory support, protekduo, right-sided heart failure

## Abstract

Choosing an appropriate mechanical circulatory support device in a timely fashion is essential during acute cardiogenic shock management. We present a case of a 52-year-old man who was admitted for elective left heart catheterization, whose procedure was complicated by right coronary artery dissection, leading to acute cardiogenic shock, and subsequently required microaxial left ventricular assist devices for both left and right mechanical circulatory support (left Impella CP and Impella RP). He went through a tumultuous course post circulatory support initiation and required various other configurations of support devices including venoveno-arterial pulmonary arterial extracorporeal membrane oxygenation (V­V-APa ECMO) and ProtekDuo. This is a single successful report of converting from venoarterial extracorporeal membrane oxygenation (V-A ECMO) to ProtekDuo in the setting of acute cardiogenic shock from right heart failure via a stepwise approach. The escalation and de-escalation of mechanical support in our patient illustrate the importance of choosing an appropriate configuration of mechanical support devices at the right time during the management of cardiogenic shock.

## Introduction

In patients with acute myocardial infarction (MI), approximately 40% develop right ventricular dysfunction, which is associated with an in-hospital mortality of 40-50% [1-3]. Moreover, 5-10% of patients with acute MI develop cardiogenic shock [3,4]. Contemporary strategies for treating patients in cardiogenic shock after acute MI include early revascularization, chronotropic and ionotropic support, optimization of pre- and after-load, and timely use of mechanical circulatory support (MCS) devices [5]. The choice and timing of MCS are largely chosen based on patient history, etiology of the MI, and practical concerns. Currently, guidelines from the American Heart Association do not specify timing or device choice to escalate or de-escalate MCS devices [6]. Although there is no published algorithm for the escalation and de-escalation of right ventricular (RV) support, choosing the correct type and combination of RV support at the appropriate time are key to success in patients with severe acute cardiogenic shock.

Of all the clinically available right ventricular assist device (RVAD) options, the ProtekDuo dual-lumen cannula is well-known for its versatility, allowing for a multitude of canulation strategies. It offers a groin-free approach to provide temporary hemodynamic support in patients with right heart failure, with the advantage of an option to add an oxygenator to convert to venovenous extracorporeal membrane oxygenation (V-V ECMO), in addition to allowing for complete mobilization and bedside decannulation [7]. Since its first clinical use in 2016, it has been used as a temporary RVAD in patients with right heart failure after MI [7]. However, its application in the hybrid circuit in an existing venoarterial extracorporeal membrane oxygenation (V-A ECMO) circuit for right heart failure has not been described. We describe the use of a duo-circuit venoveno-arterial pulmonary arterial extracorporeal membrane oxygenation (V­V-APa ECMO) with an RVAD via a ProtekDuo cannula to support a patient with severe right ventricular failure after cardiac arrest from a myocardial ischemic event. Written Health Insurance Portability and Accountability Act (HIPAA) authorization was obtained from the patient.

This article was previously presented as a medically challenging case at the Anesthesiology 2023 meeting on October 13, 2023.

## Case presentation

A 52-year-old man with a past medical history of hypertension, obesity with a body mass index of 34.6 kg/m^2^ (weight: 110.7 kg, height: 180.3 cm), and paroxysmal atrial fibrillation with pulmonary vein isolation performed six months prior arrived from an outside hospital after being placed on V-A ECMO.

A week earlier, the patient underwent left heart (LH) catheterization in a peripheral hospital for intermittent left-sided chest pain despite a normal transthoracic echocardiogram (TTE). The LH catheterization found focal high-grade stenosis in the mid-right coronary artery (RCA), in addition to mild to moderate left anterior descending (LAD) artery and circumflex artery disease. Two drug-eluting stents were placed in the mid-distal RCA, achieving 0% stenosis at the site of stenting with thrombolysis in myocardial infarction (TIMI) grade flow of II (TIMI II) distally (preintervention: 70-80%). The procedure was complicated by RCA dissection with subsequent episodes of torsades de pointes requiring multiple rounds of defibrillation and cardiopulmonary resuscitation. After intubation, return of spontaneous circulation was achieved. An Impella® CP (Abiomed, Danvers, Massachusetts, USA) was inserted via the left femoral artery in the setting of cardiogenic shock. Due to persistent hypotension, access was obtained in the right common femoral vein with a 7Fr venous sheath. Right heart catheterization with a 7Fr Swan-Ganz catheter was performed, and evidence of RV dysfunction was found. An Impella® RP was initially placed through the right common femoral vein, but the patient woke up during the procedure and dislodged the Impella® RP catheter; therefore, access was re-obtained via the left common femoral vein and re-positioned in the left proximal pulmonary artery under fluoroscopic guidance.

**Figure 1:**
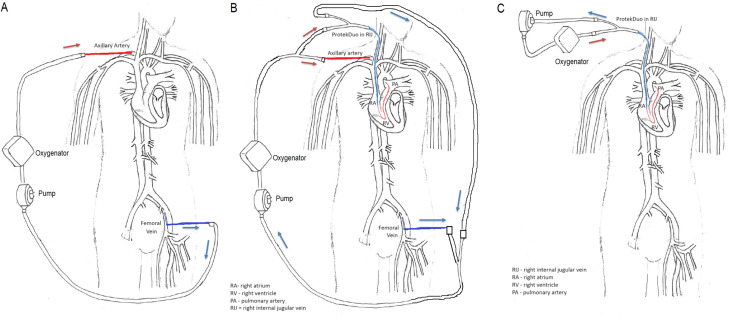
Illustration of the ECMO cannulation timeline The image was created by Zi Wei Liao, MD. A) Extracorporeal membrane oxygenation (ECMO) Day 1: venoarterial extracorporeal membrane oxygenation (V-A ECMO) circuit. B) ECMO Day 3: venoveno-arterial pulmonary arterial extracorporeal membrane oxygenation (VV-APa ECMO) circuit. A ProtekDuo cannula in the right jugular vein and a percutaneous cannula in the femoral vein drained blood from the right atrium and left femoral vein, respectively. Blood is then pumped through an oxygenator before returning to the patient via the ProtekDuo cannula to the right pulmonary artery and via a percutaneous cannula to the axillary artery. C) ECMO Day 5: veno-pulmonary arterial extracorporeal membrane oxygenation (V-Pa ECMO). The arrow represents the direction of blood flow. Blue: deoxygenated blood. Red: oxygenated blood

At the local hospital, the Impella® CP had been removed due to signs of left ventricle recovery on hospital day (HOD) 3 and leaving only Impella® RP in place for right ventricle support. Two days after the left-sided Impella® was removed, the patient acutely decompensated with increasing pressor requirements, lactic acidosis (8 mmol/L), and worsening bilateral pulmonary edema. He was taken to the cardiac catheterization lab, and it was confirmed via fluoroscopy that his right-sided Impella® became dislodged again. Given the ProtekDuo (LivaNova Inc., London, UK) cannula was not in stock at the local hospital, he was percutaneously cannulated to V-A ECMO with a 26Fr drainage canula in the left femoral vein and a 18Fr return canula in the right axillary artery, as femoral access was used for the initial left heart catheterization, prior to transferring to a tertiary care facility for further ECMO management (Figure 1A).

The patient arrived on 0.2 mcgꞏkg^-1^min^-1^ epinephrine, 0.04 units/min vasopressin, and 0.08 mcgꞏkg^-1^min^-1^ norepinephrine with ECMO flow >3 L/min to maintain mean arterial blood pressure >65 mmHg. Dobutamine 5 mcg/kg/min was added to wean epinephrine to 0.08 mcgꞏkg^-1^min^-1^. He was started on inhaled nitric oxide for selective pulmonary vasodilation at 40 ppm, given his TTE showed severely decreased RV function (pulmonary artery systolic pressure 25-34 mmHg, pulmonary artery diastolic pressure 10-12 mmHg, central venous pressure 15-18 mmHg) despite preserved left ventricle (LV) necessitating RV support. On ECMO Day 3, he was cannulated with a 29Fr ProtekDuo cannula via the right internal jugular vein (pulmonary artery systolic pressure 19-22 mmHg, pulmonary artery diastolic pressure 10-14 mmHg, central venous pressure 7-12 mmHg). The ProtekDuo cannula was then connected to his existing V-A ECMO cannulas with the use of Y-connectors, to convert to VV-APa ECMO (Figure 1B). This technique was chosen as it was deemed too risky to decannulate off VV-APa ECMO in the setting of an improving LV but significant RV dysfunction. He was subsequently decannulated off V-A ECMO on ECMO Day 5 and transitioned to V-Pa ECMO via ProtekDuo for continued right heart and lung support (Figure 1C). He became tachypneic and hypoxic when a wean from V-Pa ECMO to RVAD without an oxygenator was attempted; therefore, the oxygenator was left in place with the ProtekDuo on ECMO Day 6. Inhaled nitric oxide was weaned off on ECMO Day 7. He was extubated and weaned off all vasopressors and inotropes on ECMO Day 9. 

Once he had met our institution's "Readiness to wean criteria", which included right ventricular end diastolic pressure (RVEDP) 12-14 mmHg, mean arterial pressure (MAP)>65 mmHg, off pressors/inotropes, normal left atrial and central venous oxygenation (central VO_2_), normal kidney function, and improving liver function, weaning trials were attempted with rapid drop of RVAD flow to 2L/min for 30 minutes. A weaning trial is considered successful when RVEDP, MAP, and heart rate remain the same and post weaning perfusion parameters are normal (lactic acid, central VO_2_), as well as organ function (creatinine, liver enzymes). Two days after the patient tolerated a wean trial of RVAD flow to 2L/min, a wean trial to 1L/min then clamped off for 15 minutes was attempted with the same criteria. ProtekDuo and V-Pa ECMO were decannulated on ECMO Day 12 after he passed his wean trial and confirming significantly improved LV and RV function, reassuring that ProtekDuo could be removed. He briefly required dobutamine 2.5 mcgꞏkg^-1^min^-1^ until HOD14.

The patient was grateful to have woken up next to his wife on ECMO Day 9 when he was extubated. His immediate last memory at the time was going to sleep in the catheterization lab in an outside hospital in preparation for his elective procedure. He was discharged home on HOD17 with a view to continue outpatient cardiac rehabilitation. At five months, his right heart cardiac catheterization showed a reduced cardiac index (CI = 1.7, right ventricular systolic pressure/diastolic pressure 35/10, pulmonary artery systolic pressure/diastolic pressure 35/15), consistent with chronic systolic right heart failure. A year after his cardiac arrest, the patient continued to struggle with post-traumatic stress disorder which limited his ability to perform activities of daily living.

## Discussion

This case described the use of a multitude of RVADs and peripheral ECMO cannulation strategy from V-A, to VV-APa, and then V-Pa to treat acute right heart failure after severe biventricular cardiogenic shock in the setting of myocardial ischemia.

In the setting of a previously dislodged right-sided Impella®, a ProtekDuo cannula was the ideal choice for this patient to resume right heart support for several reasons. First of all, both of his femoral veins had been previously used for cannulation after his Impella® RP became dislodged the first time and therefore replacing the Impella® RP, which requires femoral access, was not an option. Moreover, compared with a ProtekDuo cannula, an Impella® RP would have a higher chance of dislodgement and hemolysis [8]. Similarly, although the TandemHeart RVAD (LivaNova Inc., London, UK) can be inserted percutaneously, it would require two cannulation sites, most commonly the right and left femoral veins, which were not available in this patient [9]. Surgical RVADs such as the CentriMagTM are less ideal, given that they require central cannulation via thoracotomy or sternotomy. The ProtekDuo, on the other hand, has the advantage of being a double-lumen cannula that is percutaneously inserted via the right internal jugular vein [10]. When connected to a pump, it works as an RVAD, and if connected with an oxygenator, functions as V-V ECMO [10].

Unfortunately, the local hospital did not have the ProtekDuo cannula in stock, and therefore, a decision was made to escalate his right heart support to V-A ECMO by peripherally canulating the right axillary artery and left femoral vein, in order to preserve the right internal jugular vein for future placement of the ProtekDuo after transferring to our cardiovascular intensive care unit. However, this is not an ideal strategy because V-A ECMO provides suboptimal left ventricular unloading due to increased left ventricular afterload, leading to left ventricle distension and delayed recovery [11]. Nevertheless, the patient did have an axillary return canula where the ECMO flow and the native LV outflow were additive, in comparison to a competitive outflow in a femoro-femoral configuration, which may have mitigated some of the increased left ventricular afterload associated with V-A ECMO [12].

When the ProtekDuo canula was inserted, it may have been possible to have decannulated his V-A ECMO at the same time as ProtekDuo insertion without converting to the VV-APa configuration. However, due to his recent history of biventricular cardiogenic shock, it was decided to use a step-wise approach to temporarily maintain bi-ventricular support with VV-APa ECMO, before weaning off V-A ECMO to ensure that the ProtekDuo alone was sufficient for cardiac support in this patient [13]. Although he tolerated V-A ECMO weaning trials while being cannulated with VV-APa, his respiratory status did not tolerate an oxygenator wean, and therefore, he remained on V-Pa ECMO with ProtekDuo until ECMO Day 12.

While temporary RVADs are a treatment option for a selective group of patients with acute right-sided heart failure, evidence on temporary RVAD implantation is based on small, non-randomized studies that utilized different devices [14]. Currently, a clear escalation or de-escalation algorithm has not been defined. Moreover, potential complications such as bleeding, infection, and thrombosis can occur, also there is a lack of long-term follow-up data on patients who had been supported via ProtekDuo or V-A ECMO for RV failure. 

## Conclusions

While temporary RVADs are a treatment option for a selective group of patients with acute right-sided heart failure, our case demonstrates the utility of several configurations of mechanical circulatory support including V­V-APa ECMO and ProtekDuo in the setting of severe cardiogenic shock. Importantly, the staged approach in this case not only led to successful hemodynamic stabilization and recovery, but also illustrates a potentially reproducible strategy that may be applied to other causes of RV failure, including those arising from pulmonary embolism, hypoxia, or acute decompensation in chronic pulmonary hypertension. The escalation and de-escalation of mechanical support in our case illustrates the importance of choosing the appropriate configuration of mechanical support devices at the right time. 

Our case contributes meaningfully to the literature by outlining a practical and adaptive approach to escalating and de-escalating MCS in a complex clinical scenario. Future research is warranted to better define optimal timing, selection criteria, and weaning protocols for RV-specific MCS strategies. Comparative studies evaluating long term outcomes among various RV support configurations including ProtekDuo, ECMO-based hybrids, and percutaneous options would be valuable in informing the development of future clinical guidelines.
